# Synthesis and Applications of Encapsulated Glycol-Stabilized Lyotropic Cholesteric Liquid Crystal Hydrogels

**DOI:** 10.3390/gels11060388

**Published:** 2025-05-25

**Authors:** Yan-Ting Lin, Chung-Yu Kuo, Yi Shen, Alexander V. Emelyanenko, Chun-Yen Liu

**Affiliations:** 1 Department of Materials Science and Engineering, National Cheng Kung University, Tainan 701401, Taiwan; 2 Department of Chemical Engineering, National Cheng Kung University, Tainan 701401, Taiwan; 3 Faculty of Physics, Lomonosov Moscow State University, Moscow 119991, Russia

**Keywords:** cholesteric lyotropic liquid crystal, hydrogel, sensing, color variation, crosslinking, encapsulation

## Abstract

The micro-phase segregation of two incompatible components on a nanometer scale results in a unique solvent-induced extended anisotropic arrangement. With the addition of a chiral dopant, lyotropic liquid crystals can be induced to adopt a helical structure, forming lyotropic cholesteric liquid crystals capable of reflecting incident light. In this study, to prevent fluid leakage in lyotropic materials, we encapsulated a series of hydrogel-stabilized lyotropic liquid crystals, presenting tunable structural colors visible in all directions, mimicking the color-changing characteristics of living organisms. Hydrogel scaffolds with controllable swelling behaviors were engineered by incorporating crosslinking monomers. To ensure stable integration of lyotropic liquid crystals, high-boiling-point ethylene glycol was employed as a fluid during the fabrication process. This study extensively explores the relationship between tensile force, temperature, and pressure and the color changes in lyotropic liquid crystals (LC). The results indicate that lyotropic LC membranes, stabilized by ethylene glycol and PDMS encapsulation, exhibit long-term stability, rendering them suitable for applications in temperature and pressure sensing. This approach ensures the continuous presence and stability of lyotropic liquid crystals within the hydrogel matrix.

## 1. Introduction

The helical structure of the chiral nematic phase possesses a unique ability to selectively reflect light with a wavelength matching the pitch length. The alteration in color is influenced by various factors, including temperature fluctuations, external forces, and the presence of a chiral dopant [1,2,3,4,5,6,7,8,9,10]. Natural organisms feature a type of liquid crystal wealth known as lyotropic liquid crystal (LLC) [11,12,13,14,15]. These organized lyotropic mesophases consist of amphiphilic compounds and a specific solvent [16,17,18,19]. The existence of LLC is contingent upon solvent concentration and is confined to a specific concentration range. Below the critical concentration, amphiphilic molecules (lipid or surfactant) disperse randomly without order, while, above the critical concentrations, the LLC phase becomes observable through self-assembly [20,21,22,23,24,25,26,27].

The origin of structural colors lies in the intricate interplay between incident light and periodic nanoscale structures [28,29,30,31,32]. The shift in structural coloration serves as a foundation for photonic materials, transforming external physical or mechanical stimuli into visible output signals [33,34,35,36,37,38]. Hydroxypropyl cellulose (HPC) is renowned for its spontaneous organization into a chiral nematic liquid crystal arrangement, displaying iridescent structural colors within specific critical concentrations in water. This self-assembled liquid crystal mesophase of HPC, termed a lyotropic liquid crystal, exhibits stimulus-responsive optical behaviors. Leveraging their optical properties, HPC hydrogels find applications in diverse photonic devices, including heat, humidity, and strain sensors and indicators [39,40,41,42,43]. The nanoscale micro-phase segregation of two incompatible components leads to distinct solvent-induced extended anisotropic arrangements, determined using the volume balances between the hydrophilic and hydrophobic components. Additionally, chiral polymers like chiral aromatic polyamide and synthetic polypeptide can generate a cholesteric phase within a specific concentration range in the solution. These materials showcase helical structures and reflect selective-wavelength light, earning them the designation of lyotropic cholesteric liquid crystals [44,45,46,47,48,49].

Hydrogels represent biphasic materials, constituting a blend of porous and permeable solids, with at least 10% by weight or volume of interstitial fluid being primarily composed of water. In hydrogels, the porous permeable solid forms a water-insoluble three-dimensional network of natural or synthetic polymers, coupled with a fluid that has absorbed a substantial amount of water or biological fluid. The resulting composite hydrogel, featuring the HPC element and the poly(N-isopropylacrylamide) scaffold, is termed a liquid crystal hydrogel (LCG). Notably, once dried without the fluid, the porous polymer loses its lyotropic properties [50,51,52,53,54].

NIPAM is usually used as a temperature response material through lower critical solution temperature (LCST). Based on molecular structure, N-isopropylacrylamide (NIPAM) shows strong interactions with other hydrophilic functional groups. In this study, we aim at the stabilization of LC hydrogels, and NIPAM was used for the preparation of polymer-stabilized hydrogels. To prevent the fluid from escaping in lyotropic materials, we encapsulated a series of hydrogel-stabilized lyotropic liquid crystals, showcasing tunable structural colors observable in all directions, mimicking the color-changing properties of living organisms. Hydrogel scaffolds with controllable swelling behaviors were engineered with the incorporation of crosslinking monomers. For the stable integration of lyotropic liquid crystals, high-boiling-point ethylene glycol was employed as a fluid during the fabrication process. The sustained presence and stability of lyotropic liquid crystals within the hydrogel matrix are guaranteed by this approach.

## 2. Results and Discussion

### 2.1. Optical Properties of the Prepared Lyotropic LCs

For all lyotropic liquid crystals, the interplay of temperature and concentration plays a pivotal role in determining the formation of the liquid crystal phase. In this investigation, HPC served as a solute, dissolved in DI water, leading to the development of lyotropic liquid crystal phases. The identification of cholesteric liquid crystal phases was established through polarized optical microscopy (POM).

**Figure 1:**
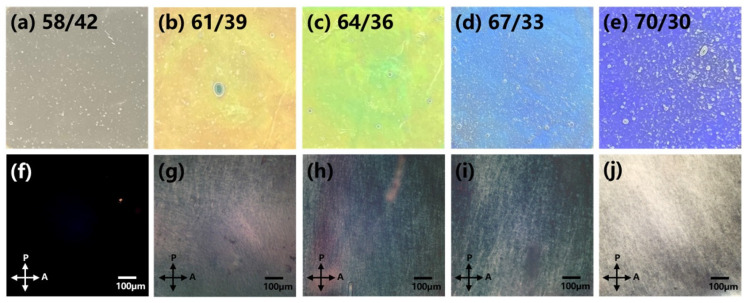
(**a**–**e**): Depict authentic sample images (OM), while (**f**–**j**): illustrate POM textures of HPC/water samples at different concentrations.

Utilizing Bragg reflection, the formation of a helical structure in the cholesteric liquid crystal manifested as a reflective interference of incident light, resulting in a colorful appearance. Figure 1a–e showcases the diverse visual aspects of HPC samples with varying water contents, with each weight ratio of HPC to water explicitly labeled in the figure. Notably, during the preparation of lyotropic sample cells, instances of the sample solution splashing onto the glass substrate led to the formation of fibrous constructions. The textures of the liquid crystal (LC) mixtures are visually depicted in Figure 1f–j. At a low HPC concentration (58/42), the POM texture appeared fully dark, indicating the presence of an isotropic phase. As the HPC concentration increased, vibrant and patterned POM textures emerged, signifying the formation of lyotropic liquid crystal phases.

**Figure 2:**
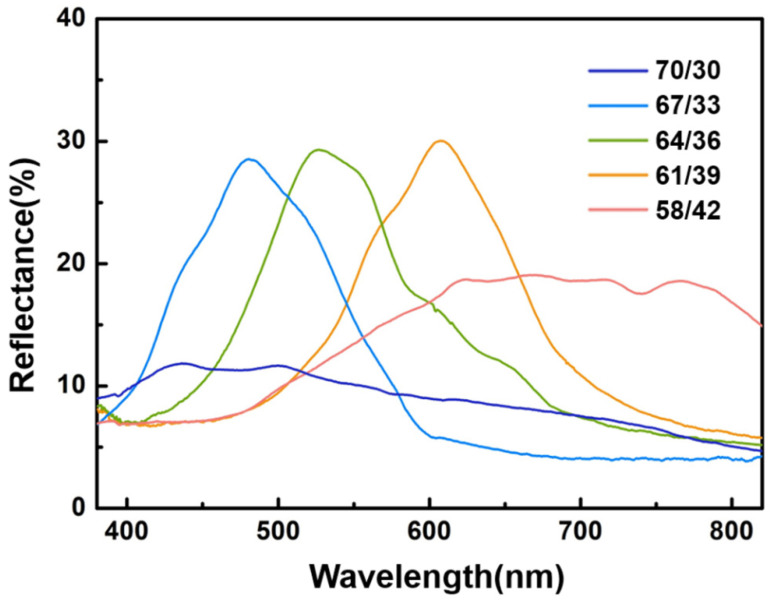
Reflection spectra of lyotropic cholesteric liquid crystal samples at varying HPC concentrations.

Figure 2 illustrates the distinct reflection bands observed in lyotropic liquid crystal samples, manifesting around 480 nm, 530 nm, and 610 nm, corresponding to blue, green, and orange wavelengths, respectively. A decrease in HPC content is associated with a red shift in the reflection bands. According to Bragg reflection theory, an increase in the concentration of chiral dopant results in a reduction in the helical pitch of lyotropic liquid crystals, leading to a blue shift.

In the case of a concentration ratio of (70/30), the lower intensity of the reflection band signifies the deformation of lyotropic helical constructions. This alteration in intensity suggests a modification in the structural characteristics of the lyotropic liquid crystal, highlighting the sensitivity of the reflection bands to changes in concentration and demonstrating the intricate interplay between composition and optical properties.

#### 2.1.1. Mechanical Properties of the LCG Films

PDMS-encapsulated LCG films were crafted in diverse shapes and thicknesses. The structural color of the manufactured LCG films exhibited a noticeable blue shift when the films underwent compression, stretching, and bending. The mechanical tests of the LCG samples are depicted in Figure S1.

#### 2.1.2. Dielectric Properties of Lyotropic LCs

Due to the dielectric properties of LCs, a bias of electric field aligns LC molecules in a specific direction. As shown in Figure S2, with the bias of an A.C. voltage (5 V) to the LC sample cell, the appearance of the sample cell changed from colorful to opaque. When it was removed from the A.C. current, recovery of the color was observed. The results indicate that the alignment of LCs was disturbed by the applied external voltage. Due to the high viscosity of the sample and polymer chain restriction, a transparent state was not observed during the A.C.-biased period. Figure S2 shows the real images of the fabricated lyotropic LC cell before and after the bias of a 5 V A.C. voltage. After removing the voltage, the recovery of the sample cell just ran at a slow speed.

Figure S3 shows the dependence of relative transmittance (λ = 632 nm) of a sample cell on the bias of A.C. voltage. After applying voltage on the N_W_15 cell, a fast response of LC molecules was observed. However, after removing the voltage, the response of the recovery of molecular alignment was relatively slower. This result is consistent with the phenomena observed in Figure S2. After removing the voltage for 90 s, the color of the cell still maintained a light green appearance. The result suggests that the reforming of the lyotropic phase was disturbed by the surrounding polymer matrix, which results in the appearance of delay. It is noteworthy that, after a long time recovering, as shown in Figure S4, the lyotropic LCs were arranged in higher order, showing deeper colorful patterns. Theoretically, a bias of voltage may force LC molecules in a specific direction; however, after removing the force, LC molecules may be strongly affected by the chiral factors, leading to the recovery of helical construction, which results in the formation of higher ordered LC arrangement. Figure S4 shows a real image of an LC sample cell before the bias of A.C. voltage, as well a depiction after the removal of voltage and stock for a long-term recovered LC sample cell. This could be due to a realignment of helical constructions forced by chiral factors, as the recovered cell had a deeper color.

### 2.2. Characterization of LCGs

#### 2.2.1. Mechanical Properties of LCGs

- Tensile Test

Investigations into two fundamental forms of tensile and compression were conducted to comprehend the mechanical properties of lyotropic chromonic liquid crystal hydrogels (LCGs). In this study, a series of LCG hydrogels was synthesized, with the components detailed in Table 1. The tensile stress–strain curve of the synthesized LCGs is presented in Figure S5. Since LCG hydrogels consist of both HPC and scaffold hydrogels, the mechanical properties depicted in Figure S5 reflect the characteristics of the fabricated LCG hydrogel. As illustrated in Figure S5, an increase in NIPAM content enhances the elasticity of the LCG. This outcome is attributed to the rise in scaffold amount and the hydrogen bonding originating from amide groups of poly(N-isopropylacrylamide) (PNIPAM). Interestingly, when compared with the N_W_15 LCG hydrogel, the ethylene glycol hydrogel N_EG_15 exhibits superior elasticity. This result suggests that HPC interacts with water and ethylene glycol in different ways, resulting in distinct mechanical properties. Compared with water, ethylene glycol has two terminal hydroxyl groups showing stronger crosslinking interaction with HPC hydrogels. Moreover, ethylene glycol is recognized as a wetting and lubricating agent. Compared to conventional polymers, the gels synthesized in this study are generally soft and mechanically weak. However, based on the stress–strain profiles shown in Figure S6, the synthesized gels can be classified into three distinct types.

**Figure 3:**
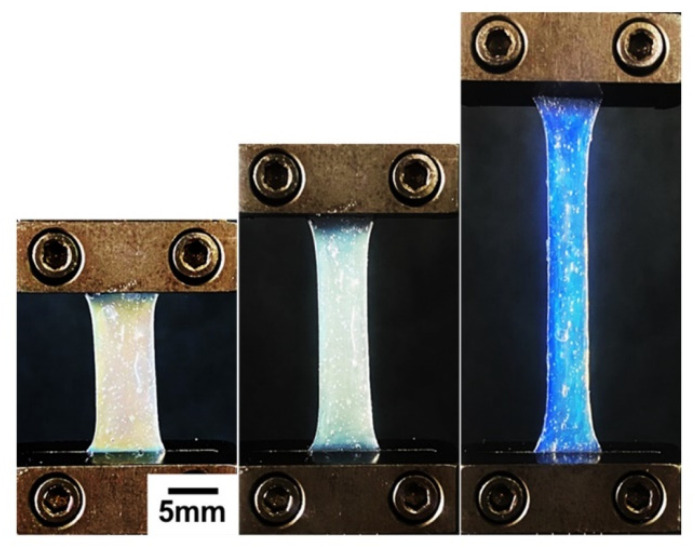
DMA test of N_EG_15 showing colorful variations.

The polyacrylamide (PAM)-stabilized LCG exhibits more elastomeric behavior, demonstrating a softer property compared to those stabilized with PNIPAM. The fracture point increases as the amount of PAM decreases, and the recovery of deformation is not optimal due to its softer nature. Based on these observations, LCG samples with a 15 wt% scaffold (N_W_15, A_W_15, N_EG_15) were chosen for further investigation. Figure 3 displays real images of the LCG sample N_EG_15 undergoing a DMA test, revealing variations of colors during stretching.

To explore the mechanical properties of the synthesized LCGs, DMA was conducted for each LCG. Figure S6 illustrates the estimated tensile Young’s modulus for both PNIPAM- and PAM-stabilized LCGs. As depicted in Figure S6a, Young’s modulus increases with the rising amount of PNIPAM. Interestingly, at the same PNIPAM content in LCGs, the ethylene-glycol-stabilized LCG exhibits a smaller value, attributed to the swelling and lubricating effects of ethylene glycol. In Figure S6b, for PAM-stabilized LCGs, the trend of Young’s modulus follows a pattern similar to that of the PNIPAM system, but the values are considerably smaller.

- Compressive Test

The compression stress–strain curves of the synthesized LCGs are depicted in Figure S7. Applying compressive stresses to LCGs with various monomers and monomer contents results in significantly different strains. Higher monomer content, as seen in N_W_20 and A_W_20, shows less compressive strain. As mentioned earlier, the synthesized LCGs comprise an HPC hydrogel and a polymeric scaffold, and the observed compressive stress of LCGs reflects the mechanical properties of the composite hydrogels. Interestingly, the compressive stress is notably influenced by the presence of ethylene glycol as a solvent. This influence may be attributed to the swelling and lubricating effects, leading to a higher compressive elasticity of the LCG.

Figure S8 presents the estimated compressive Young’s modulus at the beginning of the test for both PNIPAM- and PAM-stabilized LCGs. PAM exhibits softer behavior, requiring less stress than PNIPAM for the same degree of deformation. The solvent effect of ethylene glycol results in a greater reduction in Young’s modulus, indicating a clear difference in the elasticity of LCGs.

#### 2.2.2. Thermal Properties of the Synthesized LCGs

**Figure 4:**
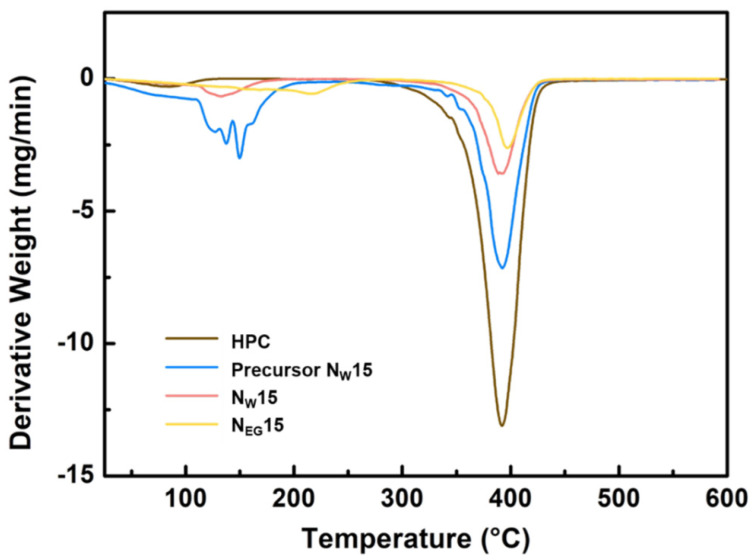
Differential TGA thermogram of precursors and LCGs.

The thermal stability of the synthesized compounds was assessed using thermogravimetric analysis (TGA). TGA was conducted under a nitrogen atmosphere at a heating rate of 283 K/min within a temperature range of 293 K to 870 K. Figure 4 presents the differential TGA graph of LCGs containing 15 wt% PNIPAM with two distinct degradation phases.

In the first inflection region depicted in Figure 4, the weight loss signifies the evaporation of hydrogen-bond-solvated water and ethylene glycol. The water-based sample N_W_15 exhibits a lower degradation temperature compared to that of the ethylene-glycol-based sample N_EG_15. This outcome may be attributed to the relatively stronger hydrogen bonding from ethylene glycol, as well as differences in boiling points. The precursor N_W_15 exhibits a broader, drastic weight loss rate than the synthesized LCG. Without polymerization, the water interacted with by the precursor components evaporates separately, resulting in broad outcomes.

In the second inflection region, the weight loss of HPC indicates the degradation of polymeric cellulose. The synthesized N_W_15 displays a lower degradation rate than the precursor N_W_15, possibly due to scaffold network inhibition. Furthermore, ethylene glycol, consisting of two hydroxyl groups, exhibits stronger interactions, leading to the lowest degradation rate at a higher temperature for the synthesized N_EG_15 LCG.

#### 2.2.3. Morphology of Synthesized LCGs

**Figure 5:**
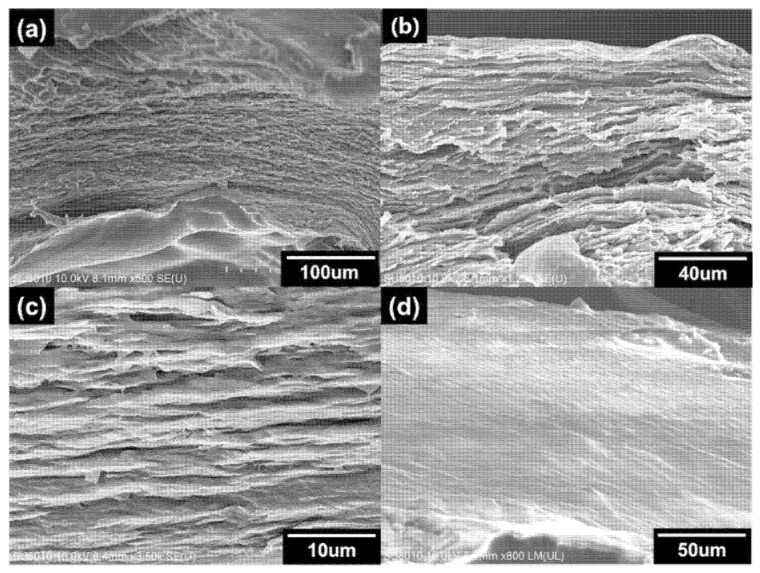
SEM image of (**a**–**c**) cross-section and (**d**) surface of LCG sample N_w_15.

The cross-section and surface of the fabricated LCGs were examined using scanning electron microscopy (SEM). The morphologies of the LCGs are presented in Figure 5, revealing layer-by-layer structures indicative of the helical construction of cholesteric liquid crystals. The pitch of the cholesteric liquid crystal is too small to be seen clearly. However, the layered structure denotes the formation of cholesteric liquid crystals. As anticipated, Figure 5a–c depicts the cross-section of the N_W_15 sample in a different scale [55]. Additionally, Figure 5d displays the smooth surface of the fabricated LCG hydrogel.

#### 2.2.4. Thermal Responsive Color Variations

**Figure 6:**
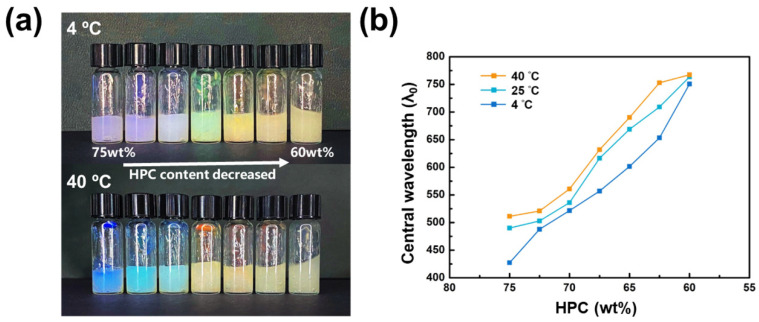
(**a**) Thermal responsive color variation of LCG precursor and (**b**) dependence of reflection central wavelength on temperature.

To investigate the temperature effect on reflection color, the LCG precursors were initially stabilized by placing them in a 4 °C refrigerator for an hour, followed by exposing the samples to a 40 °C oven for 30 min. Throughout this cycle, a red shift in the sample solution’s color was observed. Real images depicting the color variation of the LCG precursor at different temperatures are shown in Figure 6a. Figure 6b illustrates the dependence of the central wavelength of reflected light on the surrounding temperature. As the temperature increases, a red shift in color variation is observed. According to Bragg reflection, the increase in temperature leads to enhanced polymer swelling, resulting in an increased pitch and the appearance of a red shift in color.

#### 2.2.5. Stress-Induced Color Variation

The synthesized elastic LCGs display reversible deformation at room temperature, attributed to the resilience of the fibrous HPC and the network structure of the hydrogel PNIPAM. The network structure can be deformed through the application of force. In this study, lyotropic polymer HPC with a 15 wt% crosslinked NIPAM scaffold was synthesized. Scheme S1 illustrates the dependence of hydrogel network construction on the helix pitch of lyotropic liquid crystals under external force.

Applying tensile and compressive stress to the LCG induces direct deformation of the hydrogel construction, leading to the reduction and expansion of the helical pitches of the lyotropic LCG. Real images depicting color variation during stretching and compressing are shown in Figures S9 and S10. In Figure S11, N_W_15 was uniaxially stretched to 140% in length by hand. A blue shift of the LCG was observed during stretching, and a red shift was seen when the applied tensile force was released. However, solvent evaporation introduced defects, as shown in the results depicted in Figure S9. To mitigate this, ethylene glycol was used instead of water, as demonstrated in the improvement shown in Figure S10a. The evaporation of the solvent was inhibited, resulting in better elasticity without defects.

For further enhancement, PDMS was employed for the encapsulation of LCGs. Figure S10b illustrates a PDMS-encapsulated N_EG_15 LCG, which exhibits rapid recovery to the initial state and a significant increase in stretching cycles. To investigate the correlation between color variation and external force, square N_EG_15 samples were specifically prepared. Real images depicting the color variation based on external forces are presented in Figures S11 and S12.

In Figure S11a, stretching from the four corners accentuates the color variation, particularly noticeable at the corners, indicating a heterogeneous force distribution on the precursor sample. Uniaxial stretching from the upper and lower sides intensifies the color variation, especially at the corners. Figure S11b demonstrates similar heterogeneous color variations, but due to the polymer network, the color variation in the HPC content LCG exhibits more diverse and vibrant variations. Beyond the four corners, some areas concurrently show color variations, attributed to force transfer via the polymer constructions.

To further explore the color variation under stress, a 0.65 cm^2^ stamp with a pattern was prepared. Figure S12 displays real images of the color variation under stress based on the N_EG_15 precursor and polymer. Achieving the same degree of color variation requires higher pressure for polymerized N_EG_15; however, the induced color persists for a longer duration compared to the precursor sample. This outcome is attributed to the stabilization of the PNIPAM scaffold.

#### 2.2.6. Stability of LCGs

- Stretching Stability

**Figure 7:**
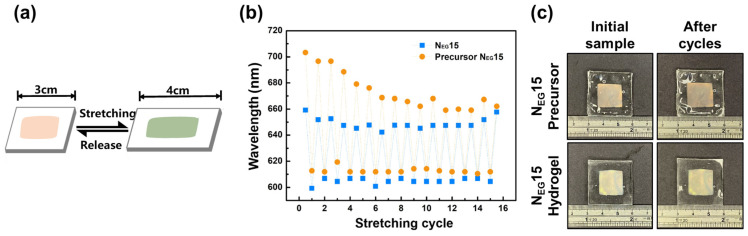
(**a**) Schematic representation of the stretching and releasing process of LCG. (**b**) Depicts the variation in wavelength, while (**c**) presents real images documenting the transformation of the N_EG_15 precursor and polymerized hydrogel across 15 stretching/release cycles.

The reversible color shift of the LCG sample was characterized by conducting stretching cycles with a constant deformation length of 1 cm, as illustrated in Figure 7a. The reflection wavelength of the extended state and the restored state was measured at intervals of 5 s. After 15 cycles, the final restored state was measured after 5 min. Initially, a significant difference was observed, with 704 nm for the precursor and 660 nm for the polymer. As illustrated in Figure 7b, the polymerized N_EG_15 sample exhibits more stable color variations compared to the precursor sample. After 15 cycles, the stress-induced color variation in both systems becomes almost the same. These results suggest that polymerized N_EG_15 is a superior candidate for the design of stress sensors. Real images of both the N_EG_15 precursor and the N_EG_15 before and after 15 stretching cycles are depicted in Figure 7c.

- Loading Stability

**Figure 8:**
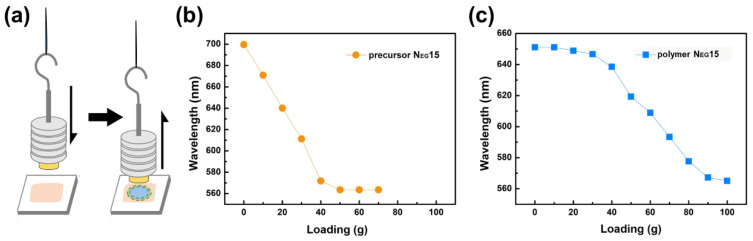
(**a**) Illustration depicting the loading test and stress sensitivity of N_EG_15. (**b**) Displays the stress sensitivity of the N_EG_15 precursor, while (**c**) demonstrates that of the polymer.

To investigate stress under specific loading conditions, the weights of various masses were attached to the stamp with a size of 0.65 cm^2^, as illustrated in Figure 8a. Figure 8b illustrates that the reflected central wavelength (λ_0_) of polymer N_EG_15 starts to decrease when 10 g loading is applied and levels off at 40 g loading. Similarly, Figure 8c depicts the dependence of the central wavelength on stress with various loadings for the precursor sample. The reflected central wavelength (λ_0_) of N_EG_15 drastically shifts down at 40 g loading and levels off at 90 g loading. Polymer-stabilized LCG films can withstand higher loadings and exhibit a more effective stress change interval.

**Figure 9:**
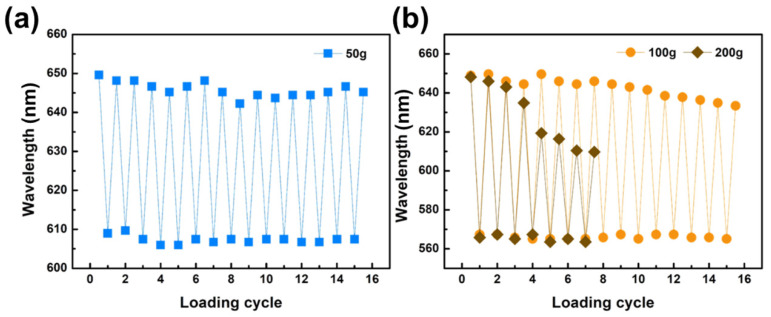
Loading cycle of polymer-stabilized N_EG_15 with (**a**) 50 g, (**b**) 100 g, and 200 g.

Loading cycles were performed on the polymer-stabilized N_EG_15 film with loads of 50 g, 100 g, and 200 g. The restored state was measured after 90 s of releasing the loading stress. The 50 g loading cycle tests are presented in Figure 9a, while the 100 g and 200 g loading cycle tests are shown in Figure 9b. After 15 cycles of 50 g loading, the color tuning of the samples is almost the same. However, in the 100 g and 200 g loading cycles, the color varies noticeably, and the reflective wavelength of the restored state decreases. These results suggest that, in the appropriate loading range, polymerized N_EG_15 can be considered for designing shape memory sensors.

## 3. Conclusions

After the solvent in the hydrogel evaporates, the hydrogel loses its functionality and may even undergo changes in its properties. To enhance the long-term stability of the hydrogel, this study employs ethylene glycol as a high-boiling-point solvent and utilizes PDMS as a protective capsule to prevent solvent evaporation, preserving the hydrogel’s characteristics. Various lyotropic LC samples were prepared in this study using solvents with different compositions to investigate the optical properties and stability of hydrogels prepared with different solvent components. By employing ethylene glycol and PDMS as high-boiling-point solvent and outer protective membrane, respectively, for lyotropic LC samples, the mechanical and optical stability of hydrogel characteristics were examined. The results indicate a significant improvement in the stability of lyotropic LC hydrogels when ethylene glycol is used as the solvent and PDMS as the outer protective membrane. The prepared lyotropic LC hydrogels can find applications in the design of temperature- and pressure-sensing devices. The findings of this study expand the feasibility of future applications of lyotropic LC in the design of flexible mechanical components and artificial sensing devices.

## 4. Materials and Methods

### 4.1. Materials

All chemical reagents and solvents used in this study were of ACS grade and were employed without further purification. Hydroxypropyl cellulose (HPC) with a molecular weight of 8000~10,000 was procured from Sigma-Aldrich (Burlington, MA, USA). N-Isopropylacrylamide (NIPAM) (99%) was purchased from Across Organics (Waltham, MA, USA). Acrylamide (AM) was purchased from Ferak Berlin (Lahnstraße, Germany). N,N-Methylenebis(acrylamide) and 2-hydroxy-2-methylpropiophenone (≥96%) were obtained from Tokyo Chemical Industrial (TCI, Tokyo, Japan). Ethylene glycol (≥99.0%) was purchased from Merck (Darmstadt, Germany). Polydimethylsiloxane (PDMS) agents were sourced from DOWSIL (Midland, MI, USA). Acetone and isopropanol (IPA) were purchased from Echi Chemical Co. Ltd. (Miaoli, Taiwan).

### 4.2. Instruments

The phase transitions of all liquid crystal mixtures were investigated with a differential scanning calorimeter (DSC, PerkinElmer DSC 7, Shelton, CT, USA) with a heating/cooling rate of 10 Kmin^−1^. Simultaneously, a polarized light microscope (POM, Nikon ECLIPSE C*i* POL, Tokyo, Japan) equipped with a hot stage (LINKAM T96-S, London, UK) was used, and the temperature scanning rate was determined as 10 Kmin^−1^. Thermal stability data were recorded under a nitrogen atmosphere at a heating rate of 10 Kmin^−1^ with a thermogravimetric analyzer (TGA, PerkinElmer TGA 7, Shelton, CT, USA). A helium–neon laser (λ = 632 nm) from Meredith Instruments (Peoria, AZ, USA) served as the light source. The mechanical properties were evaluated using a dynamic mechanical analyzer (DMA, RSA-G2, TA Instruments, New Castle, DE, USA), whereas the Fourier Transform infrared spectra were recorded using FTIR spectroscopy (FT/IR-4600, JASCO, Tokyo, Japan). The sample morphologies were imaged using a high-resolution scanning electron microscope (HR-SEM, SU8010, Hitachi, Tokyo, Japan). A UV–Vis spectrophotometer (LAMBDA 950, Perkin Elmer, Shelton, CT, USA) and a fiber optical spectrometer (SL1 with reflectance accessories, Stellar Net Inc., Tampa, FL, USA) were used. An ultrasonic cleaner (DC200H, Delta Ultrasonic, New Taipei, Taiwan) was used to clean the ITO glass substrates for dielectric property evaluations.

### 4.3. Fabrication of Liquid Crystal Hydrogels (LCGs)

Figure S13 illustrates the chemical structures utilized in this investigation. To prepare the hydrogels, a monomer mixture comprising N-isopropylacrylamide (NIPAM), crosslinker N,N-methylene bis(acryl-amide) (Bis-Am), and the hydrophilic photoinitiator 2-hydroxy-2-methylpropiophenone (HMPP) was dissolved in deionized (DI) water. Cholesteric lyotropic liquid crystal mixtures in 64 wt% were created by dissolving HPC in DI water or ethylene glycol. The detailed weight ratios of the sample hydrogels are provided in Table 1.

**Scheme 1:**
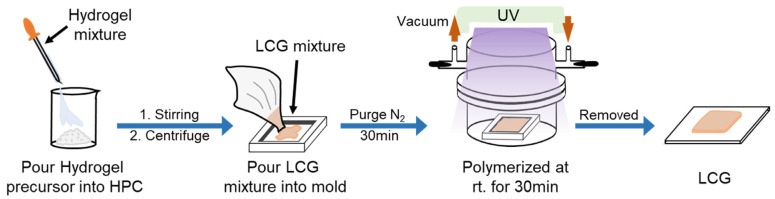
The synthetic procedures of liquid crystal hydrogel.

As depicted in Scheme 1, the monomer NIPAM, crosslinker Bis-AM, and initiator HMPP were dissolved in a specific solvent (10 g) to form a homogeneous monomer mixture. HPC was then added in a predetermined amount to the initial monomer mixture with continuous stirring. The lyotropic cholesteric gel (LCG) precursor mixture was degassed for 20 min using a centrifuge. To create a uniform gel precursor solution, the resulting mixture was poured into a PTFE mold and positioned in a sealed glass reactor for one day at 273 K. In order to eliminate oxygen, the glass reactor was purged with nitrogen for 30 min prior to polymerization. The photochemical reaction was initiated by irradiation with UV light (365 nm, 5 mW/cm^2^) for 30 min at room temperature. The synthesized LCG was subsequently stored in a 273 K refrigerator for further analysis.

### 4.4. Analysis of Dielectric Properties of LCGs

To investigate the dielectric property of the LCG hydrogels, the prepared liquid crystal hydrogels were placed between two naked ITO glasses. For the preparation, the glass substrates (2.5 cm × 2.5 cm square) were cleaned in an ultrasonic cleaner for 20 min each with a neutral detergent, DI water, acetone, and isopropanol (IPA), then dried in an oven. The liquid crystal hydrogel samples, with a thickness of 0.3 mm, were sandwiched between two ITO glasses, and copper tape was applied to the edges of each glass substrate. To evaluate the dielectric properties of the fabricated LCGs, an A.C. voltage (5 V) was applied to measure changes in transmittance. Scheme S2 shows an illustration of the testing equipment.

### 4.5. Dynamic Mechanical Analysis of LCGs

#### 4.5.1. Tensile Test

**Figure 10:**
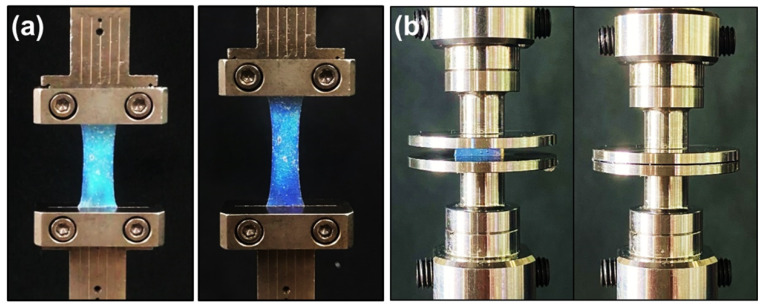
A DMA used to test the (**a**) tensile and (**b**) compressive properties of LCGs.

The LC hydrogel elastomer was fabricated in a dog-bone shape configuration (length: 2.5 mm, width: 0.5 mm), and tensile tests were conducted using TA Instruments RSA-G2 at room temperature. Prior to initiating the test, as illustrated in Figure 10a, the LCG samples were securely affixed to the tensile testing fixture, and the stretching rate was set at 0.04 mm/min.

#### 4.5.2. Compressive Test

Uniaxial compression measurements were conducted using TA Instruments RSA-G2 at room temperature in compression mode. The LCG samples were cut into square specimens with both length and width measuring 7 mm and a thickness of 2 mm. These specimens were positioned between two impermeable, unlubricated metal plates, as illustrated in Figure 10b. Subsequently, the samples were subjected to a force until reaching 100% strain, and were compressed at a constant linear rate of 0.01 mm/min.

#### 4.5.3. Morphology Observation of LCGs

The synthesized hydrogel sample was stored at 277 K in a nitrogen atmosphere for one day to attain an equilibrium swelling gel state with a helical structure. Following this, the sample was rapidly immersed in liquid nitrogen for 3 min and then subjected to high vacuum conditions for two days to stabilize the structure. The morphology of both the film surface and cross-section was examined. The prepared sample’s surface was coated with a thin layer of platinum using the sputtering method, and surface microstructures were observed via scanning electron microscopy (SEM).

#### 4.5.4. Encapsulation of LCG Films

**Figure 11:**
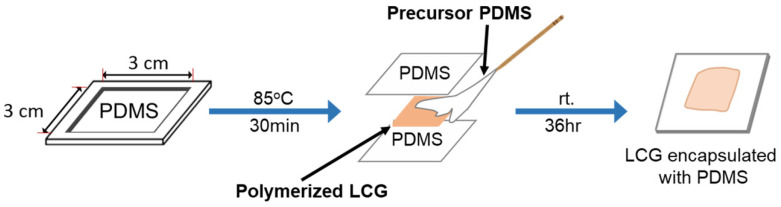
Encapsulation of the liquid crystal hydrogel film.

For the synthesis of polydimethylsiloxane (PDMS), a PDMS solution was blended with a curing agent in a 15:1 weight ratio. The resulting PDMS precursor solution was poured into a Polytetrafluoroethylene (PTFE) mold and cured at 358 K for 30 min. Figure 11 depicts the encapsulation process of the LCG film, where the prepared LCG film was positioned between two PDMS films. The periphery of the LCG film was sealed with the PDMS precursor. Subsequently, the mold was left at room temperature for 36 h to complete the curing process.

## Figures and Tables

**Table 1:** Composition of the LCGs ^1^.

	Chemicals	Monomer		Crosslinker	Solvent
Sample		NIPAM	AM	Bis-AM	DI Water/ Ethylene Glycol
**N_W_10**		1.0%	0	0.1%	DI water
**N_W_15**		1.5%	0	0.15%	
**N_W_20**		2.0%	0	0.2%	
**A_W_10**		0	1.0%	0.1%	DI water
**A_W_15**		0	1.5%	0.15%	
**A_W_20**		0	2.0%	0.2%	
**N_EG_15**		1.5%	0	0.15%	ethylene glycol

^1^ Based on hydroxy propyl cellulose.

## Data Availability

The original contributions presented in this study are included in the article/Supplementary Materials. Further inquiries can be directed to the corresponding author.

## Supplementary Materials

The following supporting information can be downloaded at: https://www.mdpi.com/article/10.3390/gels11060388/s1, Scheme S1: Schematic illustration of the reversible deformation of LCGs by forces; Scheme S2: Illustration of the dielectric properties test equipment for LCG cells; Figure S1: The mechanical test of LCG films; Figure S2: Real images of LC cell with and without applied alternating current; Figure S3: Dependence of the relative transmittance (λ = 632 nm) of the fabricated N_w_15 sample cell on bias of A.C. voltage; Figure S4: Real images of the sample cell (a) before bias of A.C. voltage, and (b) after removing of A.C. voltage and stocked for a long period; Figure S5: Tensile stress-strain curve of the synthesized LCGs; Figure S6: Calculated Young’s modulus for each (a) PNIPAM, (b) PAM stabilized samples in tensile models; Figure S7: Compressive stress-strain curve of the fabricated LCGs; Figure S8: Calculated Young’s modulus for each of (a) PNIPAM and (b) PAM stabilized samples in compressive models; Figure S9: Color variation of (a) a few hours stocked N_W_15 showing defect places, and (b) fresh N_W_15 LCG during stretching; Figure S10: Color variation of (a) naked N_EG_15 and (b) PDMS encapsulated N_EG_15 LCG; Figure S11: Real image of tension induced color variation of N_EG_15 (a) precursor and (b) polymer with 58 wt% of HPC content; Figure S12: Pressure induced color variation of N_EG_15 (a) precursor and (b) polymer via a press of stamp; Figure S13: Chemical structures of used materials for the synthesis of LCGs.
