# Supplementary material for: Synthesis and Applications of Encapsulated Glycol-Stabilized Lyotropic Cholesteric Liquid Crystal Hydrogels
Source: Gels. 2025 May 25;11(6):388. doi: 10.3390/gels11060388 (PMC12191655; doi:10.3390/gels11060388)
Supplement: Supplementary file 1 [file gels-11-00388-s001.zip › gels-3606454-supplementary.pdf]

Supporting Information

Synthesis and Applications of Encapsulated Glycol-Stabilized Lyotropic Cholesteric Liquid Crystal Hydrogels

Yan-Ting Lin, Chung-Yu Kuo, Yi Shen, Alexander V. Emelyanenkod and Chun-Yen Liu

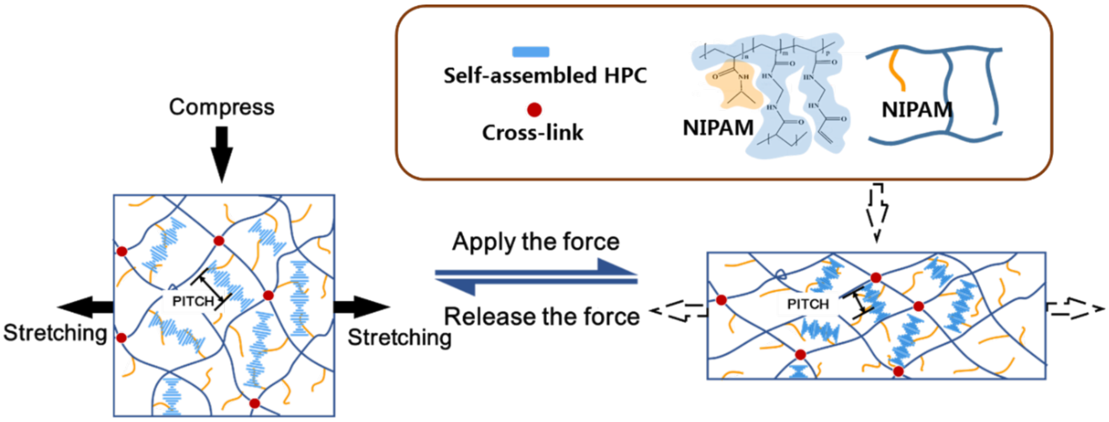

Scheme S1. Schematic illustration of the reversible deformation of LCGs by forces.

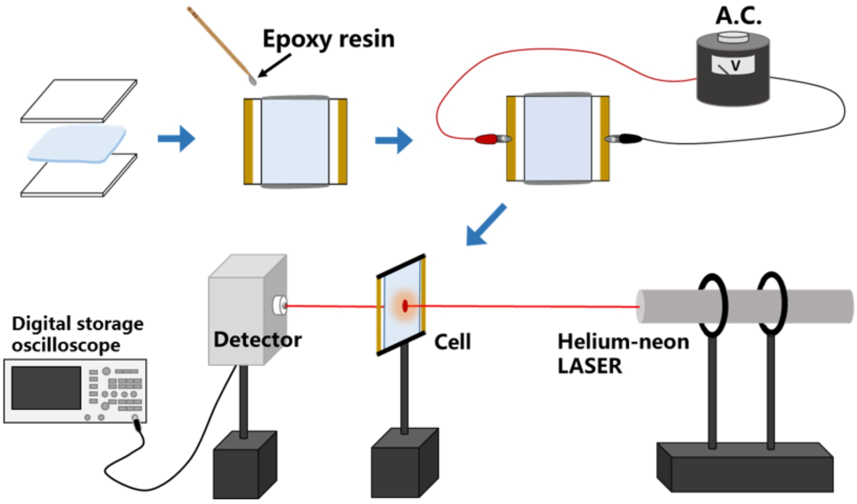

Scheme S2. Illustration of the dielectric properties test equipment for LCG cells.

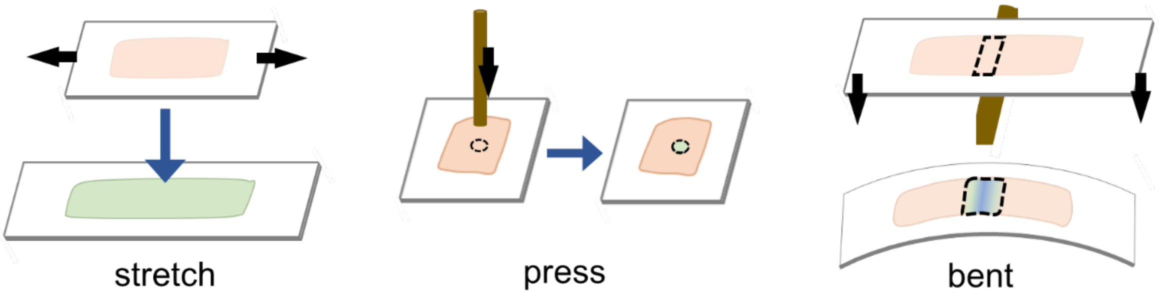

Figure S1. The mechanical test of LCG films.

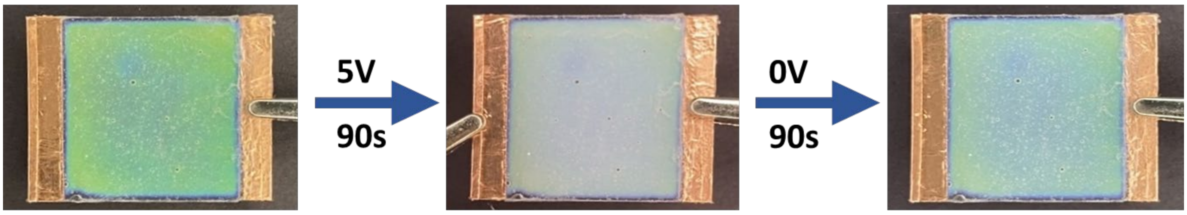

Figure S2. Real images of LC cell with and without applied alternating current.

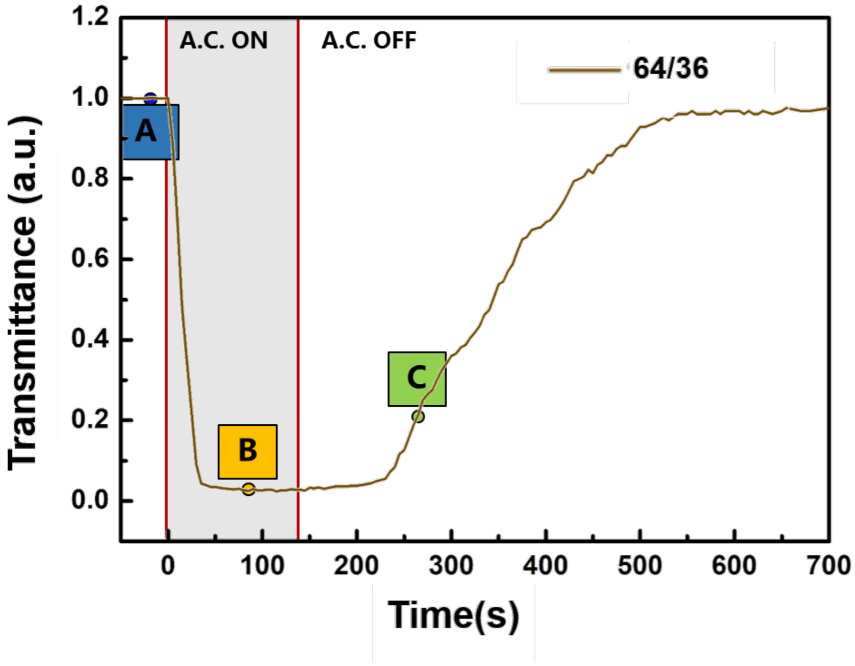

Figure S3. Dependence of the relative transmittance (λ =632nm) of the fabricated Nw15 sample cell on bias of A.C. voltage.

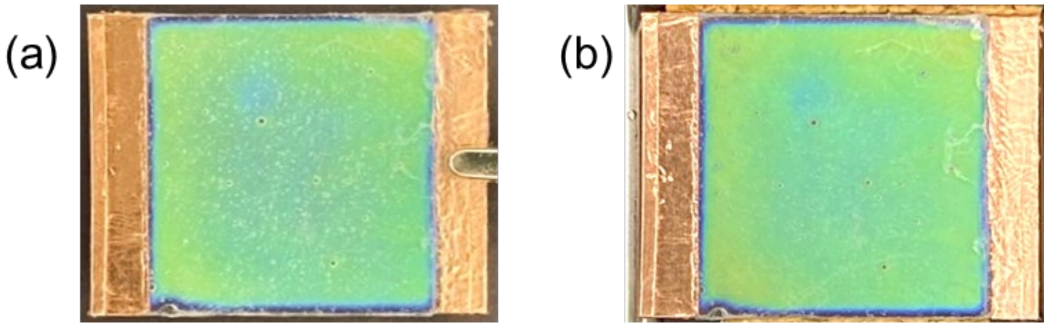

Figure S4. Real images of the sample cell (a) before bias of A.C. voltage, and (b) after removing of A.C. voltage and stocked for a long period.

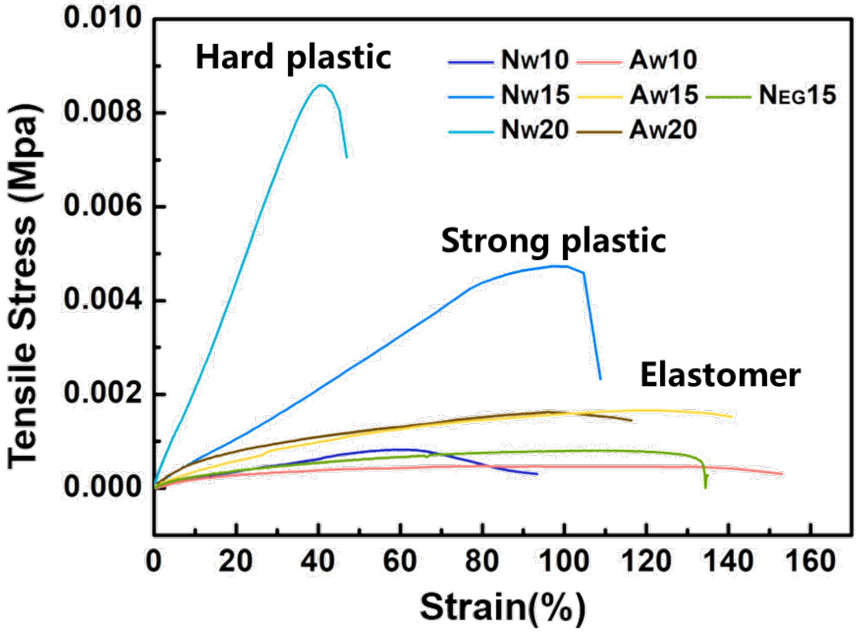

Figure S5. Tensile stress-strain curve of the synthesized LCGs.

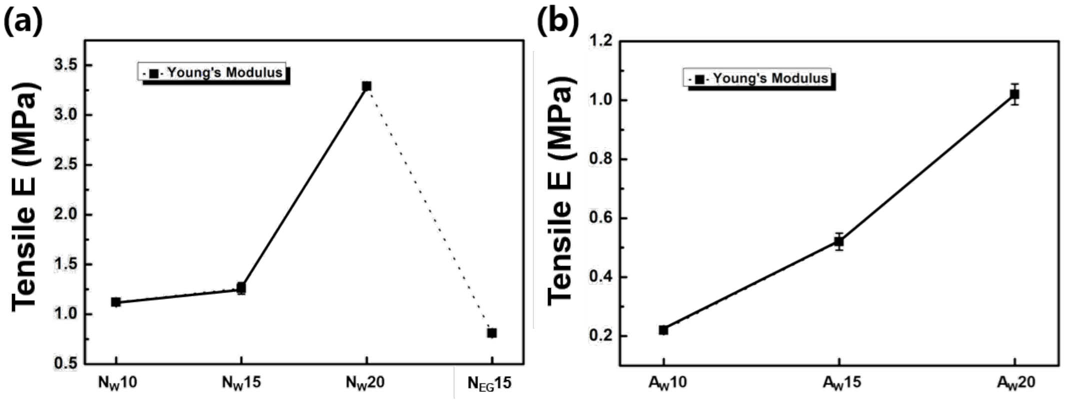

Figure S6. Calculated Young's modulus for each (a) PNIPAM, (b) PAM stabilized samples in tensile models.

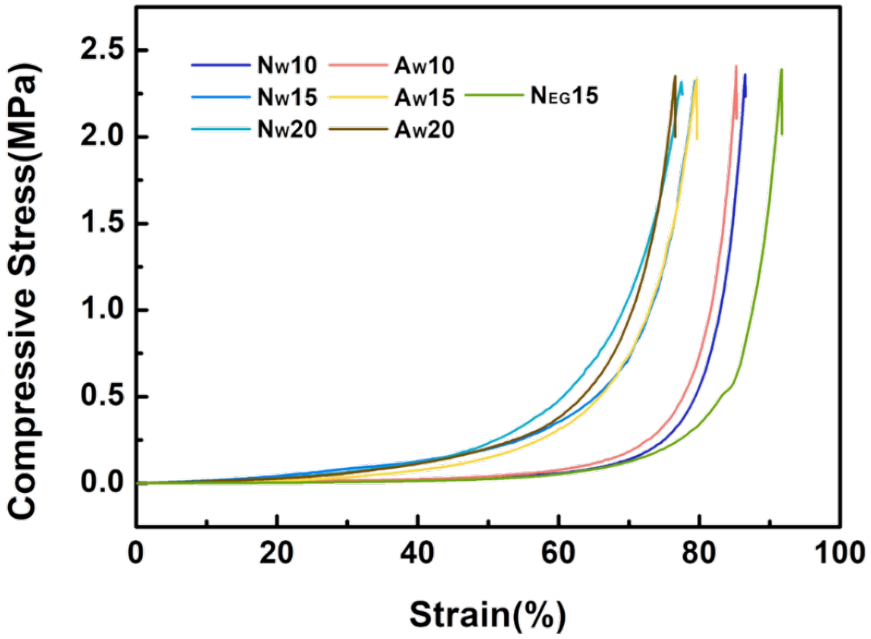

Figure S7. Compressive stress-strain curve of the fabricated LCGs.

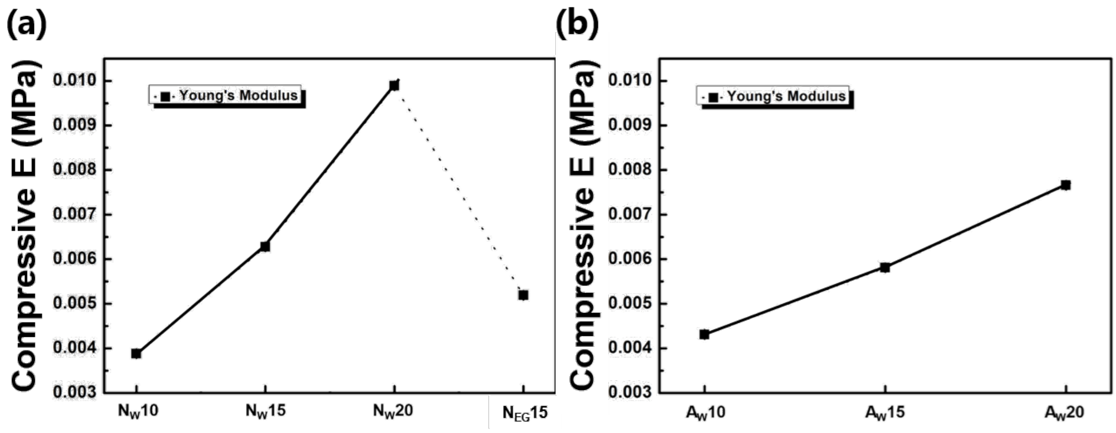

Figure S8. Calculated Young's modulus for each of (a) PNIPAM and (b) PAM stabilized samples in compressive models.

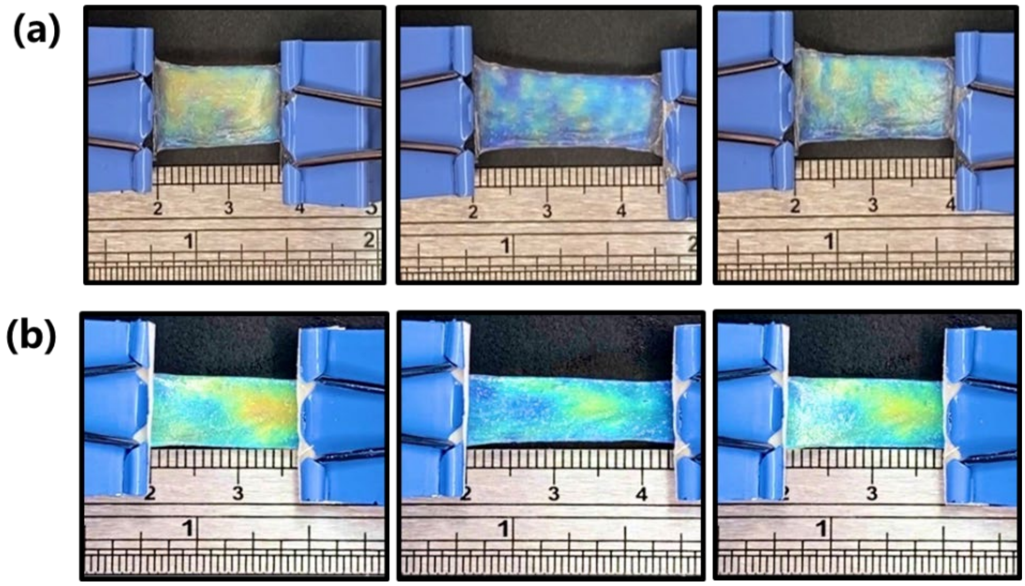

Figure S9. Color variation of (a) a few hours stocked N_W15 showing defect places, and (b) fresh N_W15 LCG during stretching.

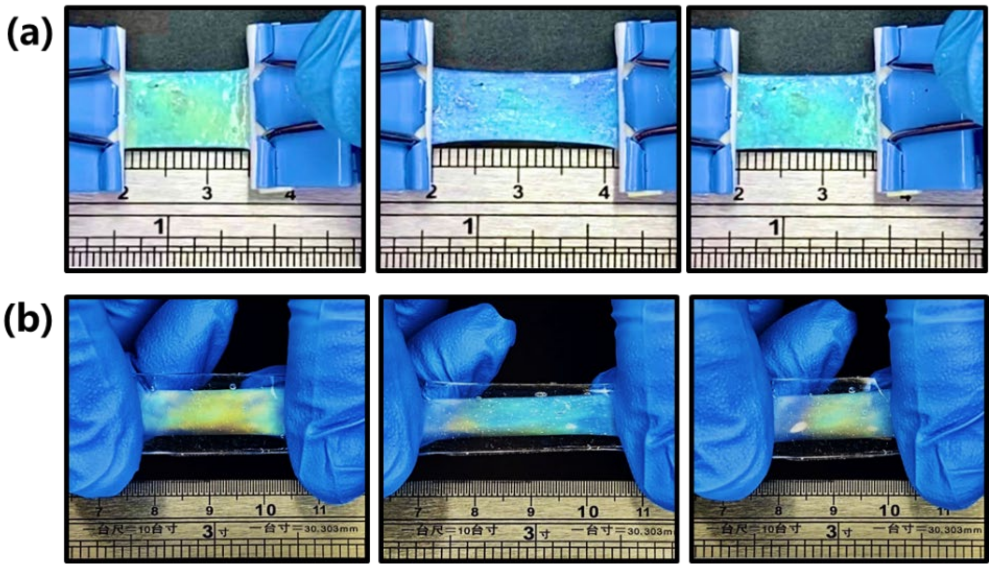

Figure S10. Color variation of (a) naked $N_{EG}15$ and (b) PDMS encapsulated $N_{EG}15$ LCG.

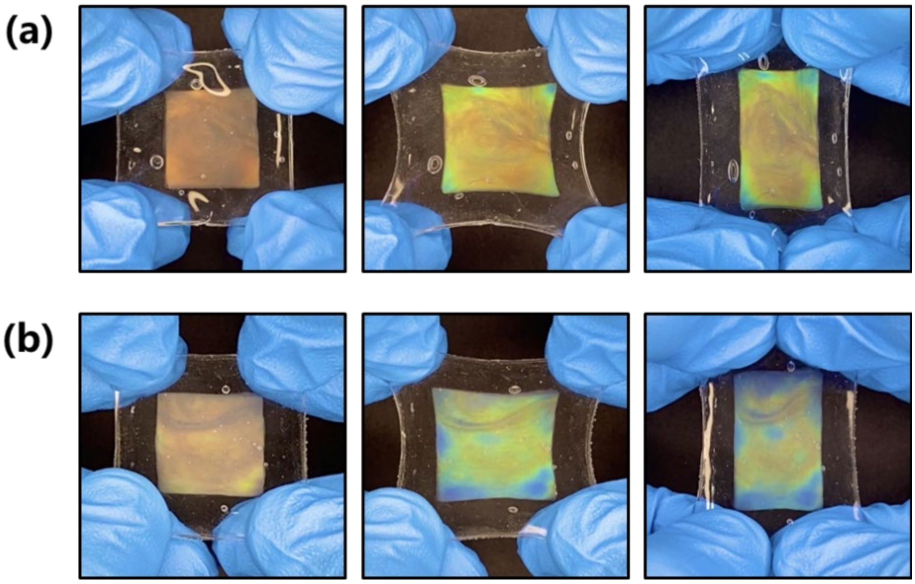

Figure S11. Real image of tension induced color variation of $N_{EG}15$ (a) precursor and (b) polymer with 58wt% of HPC content.

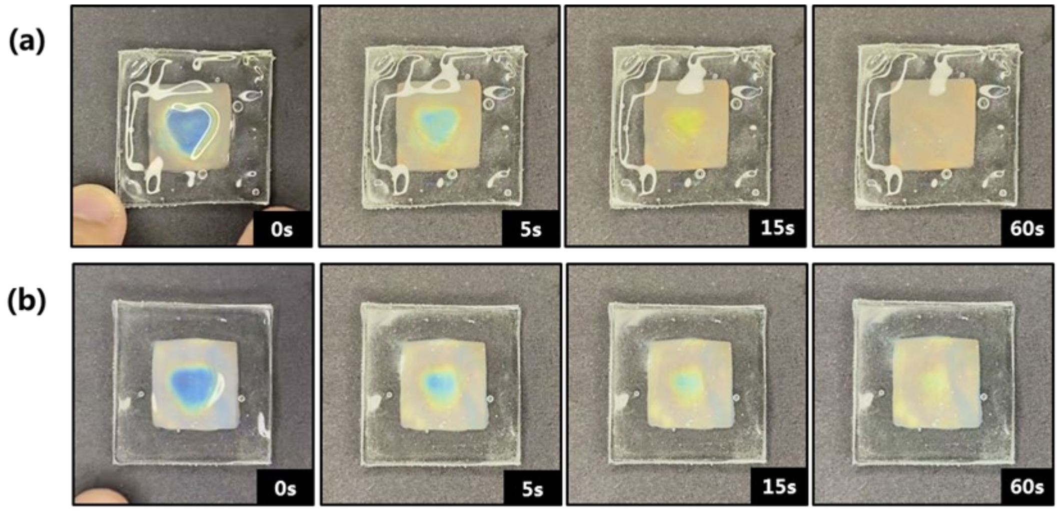

Figure S12. Pressure induced color variation of $N_{EG}15$ (a) precursor and (b) polymer via a press of stamp.

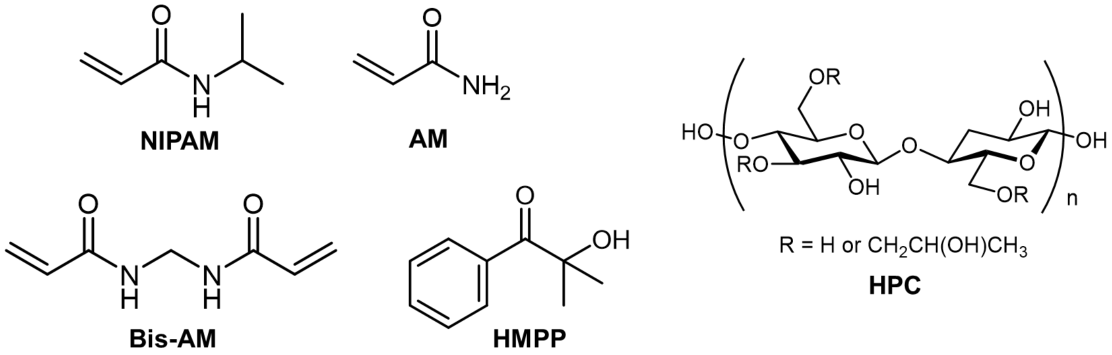

Figure S13. Chemical structures of used materials for the synthesis of LCGs.
